# Differential Effects of X-Irradiation and Cyclosporin-A Administration on the Thymus with Respect to the Generation of Cyclosporin-A-Induced Autoimmunity

**DOI:** 10.1155/1995/18495

**Published:** 1995

**Authors:** Leo J.J. Beijleveld, Jan G.M.C. Damoiseaux, Peter J.C. Van Breda Vriesman

**Affiliations:** Department of Immunology Faculty of Medicine University of Limburg P.O.B. 616 Maastricht 6200 MD The Netherlands

**Keywords:** Autoimmunity, cyclosporin A, flow cytometry, histology, rat, thymus

## Abstract

Cyclosporin A (CsA), a potent inhibitor of T-cell activation, has been shown to have several effects on thymocyte maturation, thymic stromal cells, and the generation of autoreactive T cells. In Lewis rats, the combination of lethal irradiation, syngeneic bone marrow transplantation, and a 4-week course of CsA administration causes the development of an autoimmune disease (CsA-AI) resembling allogeneic graft-versus-host disease. This occurs upon withdrawal of CsA, provided the thymus receives irradiation and is present during CsA treatment. In this study, the separate effects of irradiation or CsA treatment on thymic stromal cells and thymocytes, compared to the combinatory effects, were examined using immunohistochemistry and tricolor flow cytometric analysis.

CsA treatment causes an involution of the thymic medulla and a strong reduction of the cell number of thymocytes and stromal cells residing in the medulla. However, within the remaining medullary area, changes in cell distribution and antigen density on these cells were not observed. Irradiation on the other hand causes a strong depletion of thymocytes. The thymocyte population is recovered within 2 weeks and a cortical and medullary region can be distinguished. CsA treatment in combination with irradiation results in a strongly inhibited recovery of the medulla during CsA treatment, whereas the cortex recovers to normal size and morphology. The presence of the medullary IDC and epithelial cells is reduced proportionally to the small size of the medulla. However, the distribution of these stromal cells is normal. During the CsA administration, the thymuses from irradiated and CsA-treated rats are very similar to thymuses from CsA-treated rats. In conclusion, no changes specific for irradiation plus CsA treatment have been observed. Regarding the distribution and size of medullary stromal cells and residing thymocytes, thymuses from irradiated and CsA-treated rats hardly differ from the thymuses from rats treated only with CsA. Therefore, irradiation seems essential in the generation of CsA-AI by eliminating suppressor-cell circuits in the periphery.


*Developmental Immunology*, 1995, Vol 4, pp. 127–138
Reprints available directly from the publisher
Photocopying permitted by license only

© 1995 Harwood Academic Publishers GmbH
Printed in Singapore

### Differential Effects of X-Irradiation and Cyclosporin-A Administration on the Thymus with Respect to the Generation of Cyclosporin-A-Induced Autoimmunity

LEO J.J. BEIJLEVELD,* JAN G.M.C. DAMOISEAUX, and PETER J.C. VAN BREDA VRIESMAN

*Department of Immunology, Faculty of Medicine, University of Limburg, P.O.B. 616, 6200 MD Maastricht, the Netherlands*

Cyclosporin A (CsA), a potent inhibitor of T-cell activation, has been shown to have several effects on thymocyte maturation, thymic stromal cells, and the generation of autoreactive T cells. In Lewis rats, the combination of lethal irradiation, syngeneic bone marrow transplantation, and a 4-week course of CsA administration causes the development of an autoimmune disease (CsA-AI) resembling allogeneic graft-versus-host disease. This occurs upon withdrawal of CsA, provided the thymus receives irradiation and is present during CsA treatment. In this study, the separate effects of irradiation or CsA treatment on thymic stromal cells and thymocytes, compared to the combinatory effects, were examined using immunohistochemistry and tricolor flow cytometric analysis.

CsA treatment causes an involution of the thymic medulla and a strong reduction of the cell number of thymocytes and stromal cells residing in the medulla. However, within the remaining medullary area, changes in cell distribution and antigen density on these cells were not observed. Irradiation on the other hand causes a strong depletion of thymocytes. The thymocyte population is recovered within 2 weeks and a cortical and medullary region can be distinguished. CsA treatment in combination with irradiation results in a strongly inhibited recovery of the medulla during CsA treatment, whereas the cortex recovers to normal size and morphology. The presence of the medullary IDC and epithelial cells is reduced proportionally to the small size of the medulla. However, the distribution of these stromal cells is normal. During the CsA administration, the thymuses from irradiated and CsA-treated rats are very similar to thymuses from CsA-treated rats. In conclusion, no changes specific for irradiation plus CsA treatment have been observed. Regarding the distribution and size of medullary stromal cells and residing thymocytes, thymuses from irradiated and CsA-treated rats hardly differ from the thymuses from rats treated only with CsA. Therefore, irradiation seems essential in the generation of CsA-AI by eliminating suppressor-cell circuits in the periphery.

KEYWORDS: Autoimmunity, cyclosporin A, flow cytometry, histology, rat, thymus.

### INTRODUCTION

The fungal metabolite cyclosporin A (CsA) is a very potent immunosuppressive drug that predominantly acts on T-cell-dependent responses (Schreiber and Crabtree, 1992). It is effectively used in preventing graft rejection and graft-versus-host disease (GVHD) secondary to allogeneic bone marrow transplantation (BMT). Although CsA is also very useful in inhibiting the progression of several autoimmune diseases, surprisingly, autoimmune phenomena may rise upon withdrawal of CsA treatment under certain conditions. Rats treated with CsA after lethal irradiation and consecutive syngeneic BMT develop GVHD-like lesions after CsA administration is stopped (Glazier et al., 1983); in humans, too, similar lesions have been reported after autologous BMT (Jones et al., 1989). The crucial role of the thymus in the induction of this CsA-induced autoimmunity (CsA-AI) has unequivocally been established (Sorokin et al., 1986; Wodzig et al., 1993).

Immunosuppression with CsA not only effectively blocks the proliferation of mature peripheral T cells, but also blocks thymocyte maturation (reviewed by Ritter and Boyd, 1993). Maturation of cortical CD4 + CD8 + (double-positive) thymocytes

* Corresponding author.

into medullary CD4+ and CD8+ (single-positive) thymocytes is strongly inhibited (Gao et al., 1988; Jenkins et al., 1988; Kanariou et al., 1989). Immunohistochemical analysis of CsA-treated thymuses from rodents shows a relatively small medulla, in which the loss of medullary thymocytes is accompanied by a loss of medullary stromal cells (Tanaka et al., 1988; Schuurman et al., 1990; Ritter and Ladyman, 1991) and formation of large areas denuded of MHC class-II positive epithelial cells (Bruijntjes et al., 1993). The medullary stromal cells, consisting of epithelial cells, macrophages (Mϕ), and interdigitating cells (IDC), play an important role in negative selection of autoreactive cells. The absence or functional alteration of medullary stromal cells has been suggested to lead to the escape of possible autoreactive cells to the periphery and the development of autoimmunity (Schuurman et al., 1990; Beschorner and Armas, 1991a; De Waal et al., 1992).

Although upon CsA administration in the rat autoreactive T cells are generated (Beschorner et al., 1988b) and in the mouse the peripheral occurrence of T cells bearing self-reactive T-cell receptors, as determined by the use of mAbs recognizing specific "forbidden $V_{\beta}$s", has been demonstrated (Jenkins et al., 1988), only the combination of CsA and irradiation evokes CsA-AI in the rat. Lethal irradiation, in terms of complete bone marrow depletion, has numerous effects on the immune system, but syngeneic BMT normally leads to an almost complete recovery of the thymus (Mulder and Visser, 1988; Mulder et al., 1988; Murawska et al., 1991). Irradiation wipes out almost the whole thymocyte population, whereas the thymic stromal cells are relatively radio-resistant. Shielding of the thymus during irradiation prevents disease both in rat (Beschorner et al., 1987) and in mouse (Cheney and Sprent, 1985). It has been suggested that both whole-body irradiation, including the thymus and the CsA treatment have effects on the thymus essential for the induction of CsA-AI. Alternatively, post-CsA prevention of thymic recovery by thymectomy in unirradiated rats consistently evokes CsA-AI (Beschorner et al., 1991b). This implies that the effect of irradiation can be replaced by thymectomy after the CsA treatment, suggesting that a normal thymic microenvironment after CsA is necessary to reestablish self-tolerance through autoregulatory cells.

In our rat model, the combination of CsA and irradiation evokes CsA-AI, so this study was designed to determine the differential effects of CsA and irradiation on the thymus, as compared to the combinatory effects. The attention was focused on (1) the presence and immunohistochemical properties of the thymic stroma with special reference to the medullary IDC and epithelial cells and (2) the number and phenotype of thymocytes during and after CsA treatment. Furthermore, the disease-inducing effects of the separate treatments in combination with thymectomies were determined.

### RESULTS

### Thymus Morphology and Thymocyte Number

The thymus was morphologically assessed for the relative size of the medulla and for thymocyte density within the cortex and medulla with hematoxylin and eosin sections. The thymocyte number was determined by counting single-cell suspensions in a Bürker hemocytometer.

Total body irradiation with 8.5 Gy caused a collapse of the thymic architecture and a strong reduction of thymocytes within 48 hr. Whereas the medulla seemed only slightly affected, the cortical thymocytes had completely disappeared. One week after irradiation, there was hardly any cortex or medulla detectable, but as soon as 2 weeks after irradiation, a new cortex was formed together with a small medulla.

Two days after irradiation, the total number and viability of thymocytes were very low. During the 2 weeks following irradiation, thymocyte cell number increased from $1.2 \times 10^7$ at week 1 to $\pm 40 \times 10^7$ at week 2. Thereafter, the cell number increased much slower (Fig. 1). This thymocyte recovery was in harmony with morphological restoration of the thymus. There was little difference in total thymocyte number between SYN+ or SYN− rats, but the differences in the cortex/medulla ratio were drastic. Two weeks after irradiation, the medulla in the SYN+ thymus was very small compared to SYN− and AMC− but resembled the AMC+ thymus. As soon as 1 week after the daily administration of 7.5 mg CsA/kg, a reduction of the thymic medullary area was noticed in AMC+ thymuses. Two weeks after the initial administration, the reduction was maximal and remained the same at least until 1 week after the 4-week course of CsA-administration had been terminated, both in SYN+ and AMC+ thymuses. The cortical area recovered within 2 weeks after irradiation and seemed to be unaffected by CsA in SYN+ thymuses.

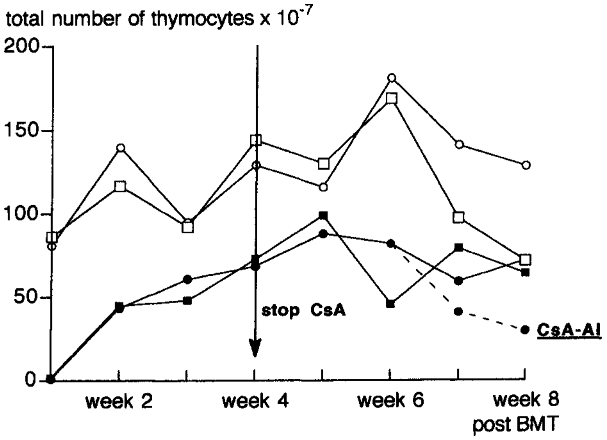

FIGURE 1. Average of total number of viable thymocytes isolated from two thymuses per group (AMC−: open squares; AMC+: open circles; SYN−: closed squares; SYN+: closed circles) at the time indicated. Cell numbers were assessed using a Bürker Hemocytometer. From week 6 and later when CsA-AI developed, two SYN+ animals free of macroscopical signs (SYN+) and two animals with clear signs of dermatitis (SYN + CsA-AI: dotted line) were assessed.

### Interdigitating Cells

We used a set of mAbs reactive with IDC in the thymic medulla. Most of these mAbs, however, are not specific for IDC, but additionally react in the thymus with epithelial cells (OX6, OX18, 1A29), Mϕ (ED1, ED8, 1A29, OX6, OX18), or thymocytes (OX18). The diffuse staining patterns of the mAbs recognizing epithelial cells or Mϕ besides IDC in the thymic medulla made it difficult to distinguish between the different cell types. Especially mAbs OX39 and OX62, which in the thymic medulla recognize specifically IDC, appeared to be very useful for the identification of this cell type.

As mentioned before in SYN+ rats, the thymic architecture was perturbed by irradiation, and the medulla recovery was strongly inhibited by the CsA treatment. As in SYN− thymuses, the effects of irradiation were very drastic; 1 week after irradiation a clearly detectable medulla was absent. Although the IDC antigens were present, it was impossible to determine whether it concerned cell debris or viable cells. Morphologically, normal cells expressing OX6, OX18, OX39, or OX62 were not detectable in the medulla. However, in SYN+ rats, a small medulla could be detected as soon as 2 weeks after irradiation (Fig. 2). The medulla of SYN+ thymuses was of comparable size as in AMC+ thymuses. OX62-positive cells and OX39-positive cells were present in a normal distribution in the medulla, as was the expression of MHC class I and II, ICAM-1, ED1, and ED8. Therefore, the expression of these markers is very indicative for the presence of IDC in SYN+ thymuses, as well as AMC+ thymuses from 2 weeks after irradiation onwards. In SYN− thymuses as soon as 2 weeks after irradiation normal distribution and expression of medullary IDC antigens was seen (Fig. 2).

A partial recovery of the medullary size in SYN+ and AMC+ thymuses was observed at 2 weeks ater cessation of the CsA treatment together with a recovery of medullary stromal cells expressing ED1, ED8, OX6, OX18, OX39, and OX62.

### Thymic Epithelial Cells

A panel of mAbs recognizing thymic epithelium was used to examine different subsets of epithelial cells. The mAbs HIS37 and RCK 102, both pan epithelial cell markers in the thymus, visualized three different areas with regard to the presence and morphology of epithelium: (1) the medulla with epithelial cells most abundantly present in the corticomedullary region, (2) the cortex with the fine reticular epithelium, and (3) the epithelial-free areas closely located to the capsule surrounding the thymus and the protruding septae. Furthermore, mAbs HIS38 and ED19 recognize cortical epithelial cells, whereas HIS39 and ED20 are specific for medullary epithelium. The epithelial cells are also detected by the earlier mentioned mAbs OX6, OX18, and 1A29.

In SYN+ rats, the cortical restoration seemed normal. HIS38 as well as ED19 clearly showed the cortical area, which was very extensive after 2 weeks. However, a combination of changes in thymic architecture was observed (see Table 1). Epithelial-free areas (EFA) close to the septae and capsule became very flat. Furthermore, after 2 weeks, vast areas void of epithelium developed surrounding the medulla. The medulla remained relatively small as determined by the medullary epithelium markers HIS39 and ED20. Thymuses from SYN+ rats showed no difference in the epithelium cell pattern compared to AMC+ thymuses at 2 weeks after irradiation, except for the absence of the flat EFAs close to the septae and capsule in the AMC+ thymuses. Overall, these changes were especially clearly visualized by the mAb OX6, recognizing MHC class II present on epithelial cells, Mϕ, and IDC (Fig. 3). In SYN− rats, irradiation damage seemed restored within 2 weeks. Typically, the EFAs close to the septae and

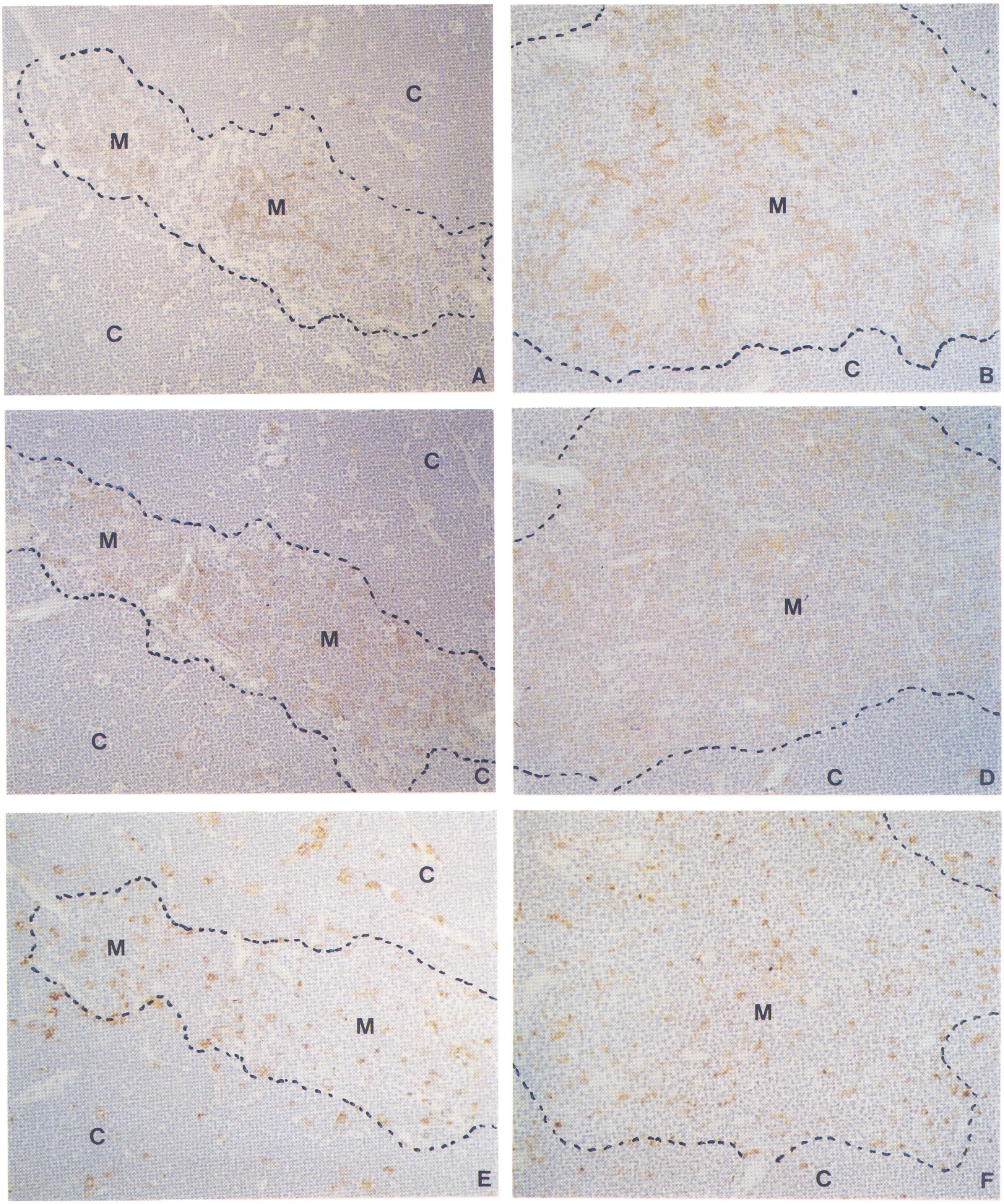

FIGURE 2. Thymuses from SYN+ (A, C, and E) and SYN− (B, D, and F) rats isolated at two weeks after irradiation and BMT. Cortex-medulla border is indicated by a dashed line; M: medulla; C: cortex (A and B) IDC as recognized by mAb OX62; (C and D) IL-2 receptor present in the medulla on IDC as recognized by mAb OX39; (E and F) Macrosialin, present in Mϕ and IDC as recognized by mAb ED1. See Colour Plate VII.

TABLE 1

| | Thymocyte Numbers (Fig. 1) | Relative Amount TCRαβ-high cells (Fig. 4) | Septae-related EFA | Medullary-related EFA |
|---|---|---|---|---|
| AMC − | Normal; steady | Normal; about 10% | Normal | Absent |
| AMC + | Normal; steady | Decreased until week 3, slow recovery | Normal | Present |
| SYN − | Decreased, slow, and incomplete recovery | Normal from week 2 | Flat | Absent |
| SYN + | Decreased, slow and incomplete recovery | Decreased until week 3, slow recovery | Flat | Present |

capsule were very flat as in SYN+ thymuses, but the epithelial-free areas around the medulla were not observed.

### Macrophages

The presence of macrophages was examined with mAbs ED1 (monocytes, Mϕ, and IDC) and ED2 (cortical Mϕ).

In SYN+ and SYN− thymuses 48 hr after irradiation in the cortex, the relatively radio-resistant Mϕ were packed very densely against each other due to the disappearance of cortical thymocytes. As soon as 2 weeks after irradiation, ED1- and ED2-positive Mϕ were normally distributed throughout the cortex, both in SYN+ and SYN− thymuses. In AMC+ thymuses ED2 distribution in the cortex was not disturbed by CsA. ED2-positive cells never were observed in the medulla, as identified by the localization of the morphological difference between cortical and medullary thymocytes, in any of the thymus sections of all four groups studied. In the EFAs surrounding the medulla, ED1- and ED2-positive cells were present in a normal distribution, comparable to the ED1 and ED2 distribution in the cortex. The distribution of ED1-positive Mϕ in the medulla was normal 2 weeks after irradiation and seemed unaffected by the CsA treatment.

### Thymocytes

The thymocytes have been studied with OX19, R73 (both expressed low in the cortex and high in the medulla), HIS45 (specific for medullary thymocytes), OX8, and W3/25 (in the cortex, all thymocytes are positive, whereas in the medulla, approximately two-thirds are W3/25-positive and one-third is OX8-positive). The antigen expression recognized by these mAbs was not changed in any of the observed thymic sections at any time. The only difference was observed in the distribution. In SYN+ thymuses 2 weeks after irradiation, a normal cortex and a small medulla could be detected in AMC+ thymuses. In the epithelial-free areas in the vicinity of the medulla, as formed by the CsA treatment, and in the epithelial-free areas close to the septae and capsule, the thymocytes had a cortical phenotype. In SYN− and AMC− thymuses, the distribution was normal. Overall, the distribution described was according to the stromal-cell distribution defining cortex and medulla. After cessation of the CsA treatment, both SYN+ and AMC+ thymuses gradually recovered as stated before, and the distribution of cortical and medullary thymocytes resembled the normal situation. At the onset of disease, a disturbance in thymic morphology was observed affecting cortex and medulla (as described by Beschorner et al., 1988a). This was associated with a reduction of thymocyte number (Fig. 1) and a decrease of thymic size.

By tricolor flow cytometry, we studied the thymocyte populations with respect to the antigens CD4, CD8, and TCRαβ. In SYN+, the number of TCRαβ-high positive thymocytes remained very low during CsA administration (Fig. 4), whereas in SYN− the strong depletion of the thymocytes was overcome as soon as 2 weeks after irradiation (Fig. 4). In AMC+, the TCRαβ-high positive thymocytes also diminished upon CsA administration (Fig. 4). Tricolor analysis showed that in SYN+ and AMC+ thymuses, the medullary CD4+TCRαβ-high and CD8+ TCRαβ-high thymocytes disappeared, whereas the cortical CD4+CD8+TCRαβ- intermediate thymocytes were unaffected (Fig. 5). After the CsA treatment was stopped, the number of CD4+ TCRαβ-high and CD8+TCRαβ-high cells started to normalize in SYN+ and AMC+ thymuses. As CsA-AI developed, the total number of thymocytes was reduced, but the relative number of CD4+ TCRαβ-high and CD8+TCRαβ-high cells remained the same. All these changes in thymocyte

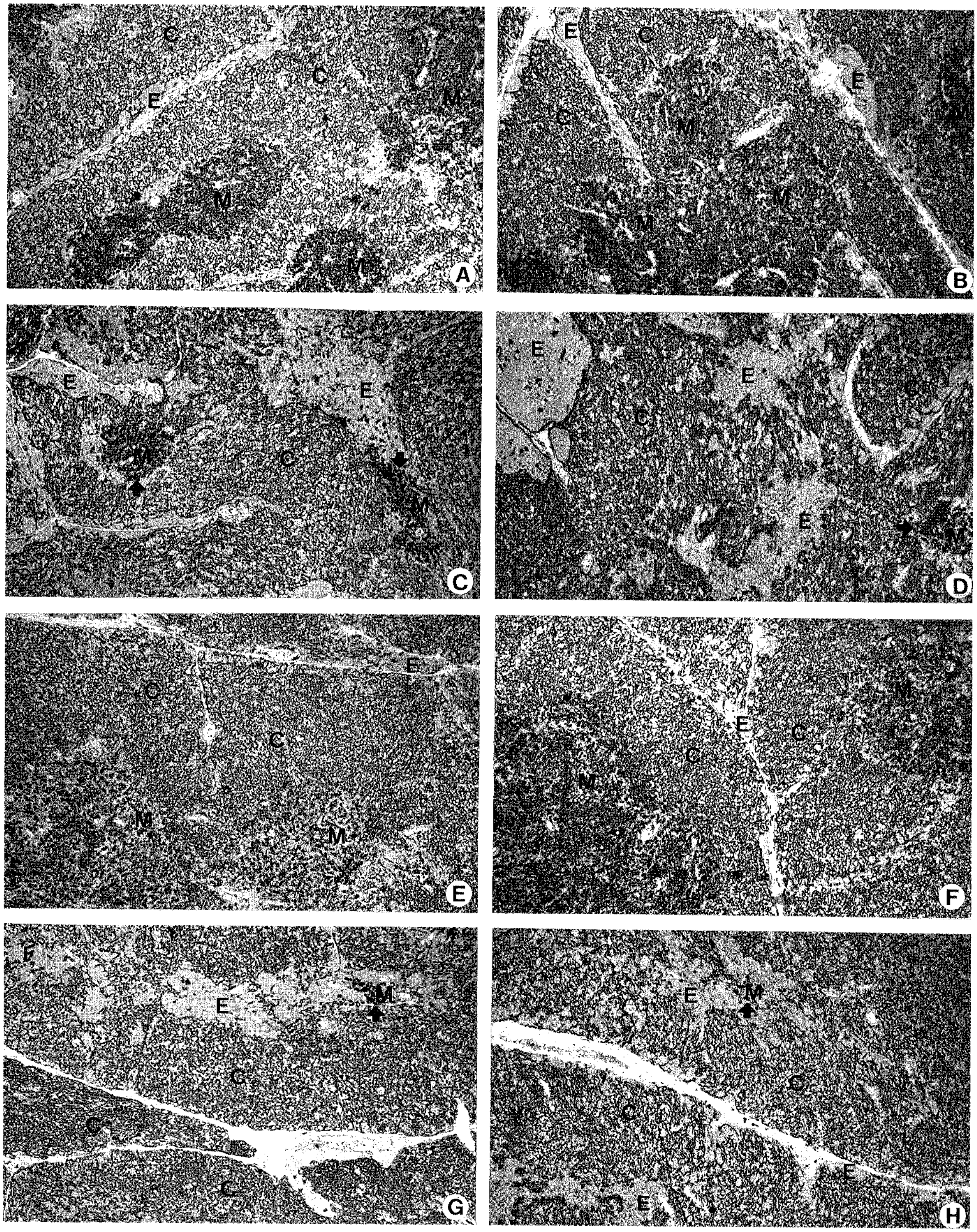

FIGURE 3. Thymuses from AMC− (A, B), AMC+ (C, D), SYN− (E, F), and SYN+ (G, H) rats at 2 and 4 weeks after initiation of the experiment. Sections were stained with mAb OX6 recognizing MHC class II (RT1B) present on epithelial cells and IDC. (A, C, E, and G) Week 2; (B, D, F, and H) week 4. E: epithelial-free areas void of MHC class II; C: cortex with fine reticular epithelium; M: medulla with IDC and epithelium. In (C and D) (AMC + ) and (G and H) (SYN + ), arrows are indicating the small medullary remnants.

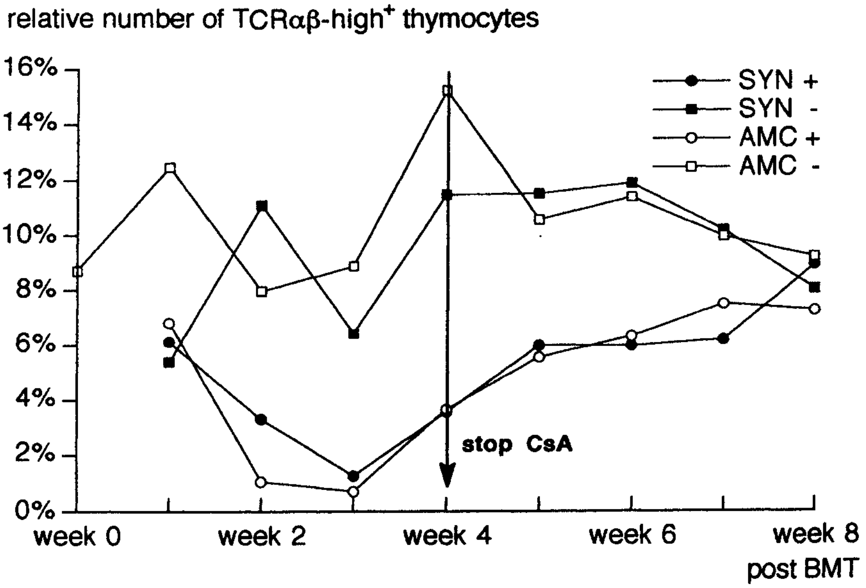

FIGURE 4. Relative number of TCRαβ-high thymocytes. Every week thymocytes were assessed for their expression of CD4, CD8 and TCRαβ using tricolor flow cytometry. By using a life gate, 10,000 cells per sample were analyzed. Per group, two thymuses were analyzed and the average relative amount of TCRαβ-high positive thymocytes is shown. AMC- thymuses were assessed from week 0, thymuses of AMC+, SYN- and SYN+ rats were tested from week 1 until week 8.

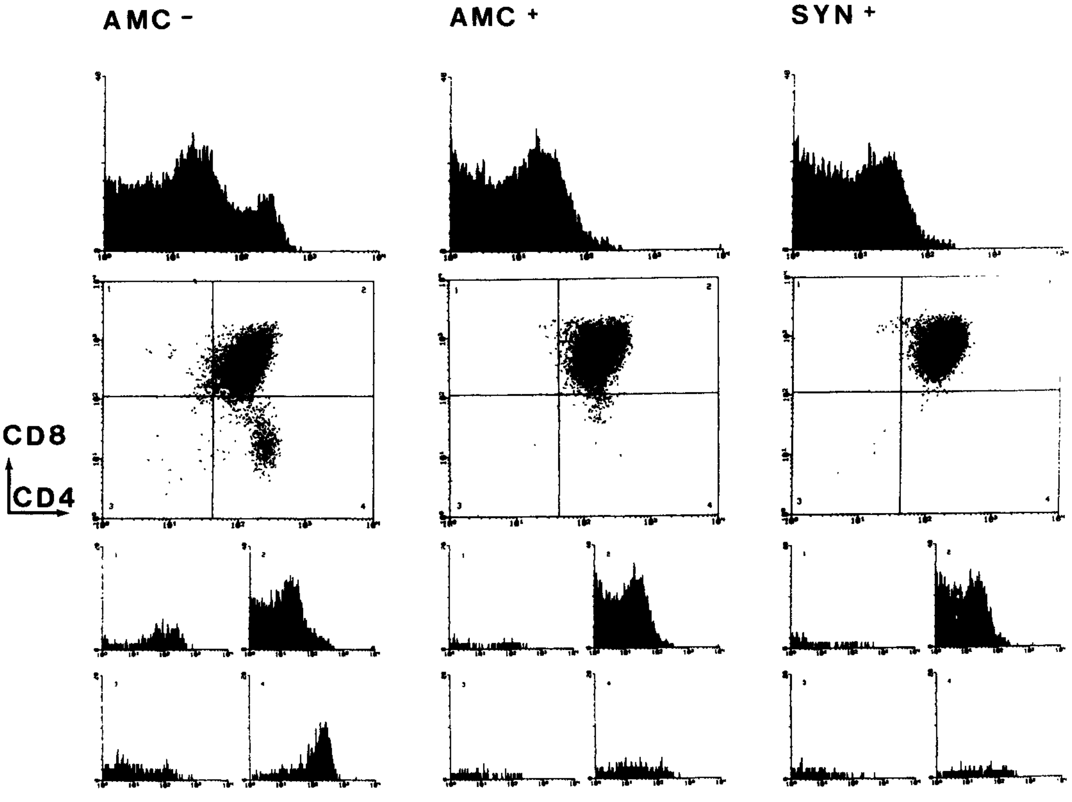

FIGURE 5. By tricolor flow cytometry, the CD4, CD8, and TCRαβ expressions of thymocytes, isolated from thymuses at week 4, were assessed. By using a life gate, 10,000 cells per sample were analyzed. The first column shows the analysis of a control thymus (AMC- ); in the second column, a CsA-treated thymus (AMC+) is analyzed., and in the third column, a thymus from an irradiated and CsA-treated rat (SYN+) is analyzed. In the upper panels, the total expression of TCRαβ is shown. The middle panels show the dot plot analysis of CD4 (FITC) and CD8 (PE) for the same thymocyte populations. Per quadrant, the TCRαβ expression is depicted in the lower panels as CD4-CD8+ in quadrant 1, CD4+CD8+ in quadrant 2, CD4-CD8- in quadrant 3, and CD4+CD8- in quadrant 4. Notice the presence of TCRαβ-high positive cells in quadrant 1 and 4 of the AMC- thymus and the absence of these cells in the AMC+ and SYN+ thymuses due to the strong decrease of CD4 and CD8 single-positive thymocytes, rather than not expressing TCRαβ by these cells.

population were in accordance with the changes observed by immunohistochemistry.

### The Effects of Thymectomies Performed After Irradiation or CsA treatment

To evaluate the effects of an abrogated recovery after irradiation or CsA treatment only, thymectomies were performed. Because the presence of the stromal microenvironment recovers in the second week after irradiation, the possible generation of autoreactive T cells during this period was evaluated by thymectomies, preventing subsequently the generation of regulatory T cells in the thymus. At days 7, 10, and 14 after irradiation and consecutive BMT each time, three SYN− rats were thymectomized and observed daily for the development of CsA-AI. None of these animals developed macroscopical signs of CsA-AI within a period of 3 months after thymectomy.

In order to test the alternative hypothesis that the effect of irradiation can be replaced by thymectomy after the CsA treatment, two groups of 12 AMC+ rats were thymectomized at day 28 directly after a continuous course of CsA administration. Again, no macroscopical signs of CsA-AI developed within a period of 3 months after thymectomy.

### DISCUSSION

The induction of CsA-AI requires the combination of lethal irradiation and syngeneic bone marrow reconstitution and the treatment with CsA for a given period. Early after irradiation, the thymus morphology is very much perturbed. Because the thymocyte population has almost completely disappeared, stromal cells are densely packed. Therefore, the presence and distribution of specific stromal cell types are difficult to determine immunohistochemically. Two weeks after irradiation the thymus is repopulated with thymocytes and a morphologically distinguishable cortex and medulla are formed. Whereas the cortex morphology and size are normal, the restoration of the medulla is strongly inhibited by the CsA treatment. The presence of the medullary IDC and epithelial cells is reduced proportionally to the small size of the medulla; however, the distribution of these stromal cells in the shrunken medulla is normal.

The effects of CsA on the thymus are still obscure. CsA causes a strong involution of the thymic medulla and a reduction of IDC in a dose-dependent manner (Damoiseaux et al., 1993). There is increasing evidence that CsA interferes with the early steps in T-cell activation by inhibiting the transcription of genes involved in cytokine production (Schreiber and Crabtree, 1992). Similar events occur in the maturation steps from cortical double-positive thymocytes into single-positive medullary thymocytes. A cascade of activation events is supposed to be essential in which several cytokines are produced (reviewed by Ritter and Boyd, 1993). It has been shown that the medullary stroma is highly dependent upon the presence of TCRαβ-high thymocytes (Shores et al., 1991; Thomson, 1992). A cytokine-driven network of factors produced by stromal cells and thymocytes might be very important for the maintenance of the medulla. The development of medullary IDC probably is dependent on the production of cytokines, such as GM-CSF and IL-2. Therefore, an effect on thymocyte maturation probably also involves an influence on IDC maturation and turnover. On the other hand, the thymocytes are highly dependent on the stromal cells for their selection. It has been suggested that the absence of stromal cells in CsA-treated thymuses is responsible for the leakage of autoreactive cells to the periphery. In this study, we showed that the antigenic density and distribution of these stromal cells are not disturbed. Thus, the role of CsA in inhibiting negative selection is not explained by this activity of CsA. It has been described that CsA also inhibits apoptosis (McCarthy et al., 1992; Yasutomi et al., 1992). Therefore, it seems likely that thymocytes that are no longer allowed to become activated due to the presence of CsA escape apoptosis and leave the thymus unselected. Although CsA treatment causes the formation of "forbidden" possible autoreactive T cells (Jenkins et al., 1988; Bryson et al., 1991), CsA treatment alone is not sufficient to cause disease (Beschorner et al., 1991b).

After irradiation and BMT, the presence of the thymus is essential during the first 2 weeks of CsA administration for the development of CsA-AI (Sorokin et al., 1986; Wodzig et al., 1993). During this period, the effects of irradiation must be overcome, and a strong influx of thymocytes is observed. The presence of medullary IDC is hard to determine and it is tempting to suggest that a lack of negative selection might occur during this period due to the absence of IDC. However, IDC are relatively radioresistant (Murawska et al., 1991) and thymectomies of SYN− rats at 7, 10, or 14 days after irradiation and BMT did not induce autoimmune phenomena. Hence, the T cells that mature early after irradiation in SYN− rats do not form an autoreactive population capable of inducing autoimmunity as they do in SYN+ rats.

Our data show morphological changes in thymic architecture, especially in the distribution of cortex and medulla. However, it seems that there are no essential differences found in thymuses from irradiated and CsA-treated rats compared to CsA-treated rats, regarding the presence of thymic stromal cells. Cells essential for thymic selection are present, and the phenotype of the stromal cells and thymocytes is normal. At the onset of disease, the thymus seems to be one of the organs affected in CsA-AI. Thymus morphology shows that this effect probably is not mediated by corticosteroids as induced by a stress response, but that the thymus is one of the target organs of CsA-AI (Beschorner et al., 1988a). Although we know that the thymus is allowed to recover after irradiation, albeit in an altered fashion due to CsA administration, very often no thymic remnants are found in the late acute phase of disease. In SYN+ rats, thymectomies performed directly after the cessation of CsA administration do not influence the course of disease, indicating the essential role for the thymus early after irradiation during CsA treatment, but not in the period thereafter. Inability to recover after CsA cessation, however, is not an explanation for development of disease as stated by others (Beschorner et al., 1991b), because in our study thymectomies performed directly after the cessation of CsA treatment in AMC+ rats do not cause CsA-AI. Because irradiation is essential to evoke CsA-AI, it is likely that there is an additional effect on the peripheral immune system that normally suppresses effectively autoreactive T cells leaking from the thymus during CsA treatment. The role and characteristics of this peripheral counterpart are under current investigation and may elucidate the contributions the thymus provides in the induction of CsA-AI.

### MATERIALS AND METHODS

### Animals

Female, specific pathogen-free Lewis (LEW, $RT1^l$) rats were used. The animals were obtained from our own breeding colony and were fed ad libitum. Animals were 6 weeks of age at the start of the experiment. For the collection of the thymus and bone marrow, rats were killed by cervical dislocation under ether anesthesia.

### Protocol for the Induction of CsA-AI

The experimental protocol has been described before (Bos et al., 1988). In brief, rats were given 8.5 Gy at 0.5 Gy/min using a Röntgen irradiator (Philips MG320, Hamburg) 1 day prior to syngeneic BMT. Bone marrow was collected from tibias and femurs in balanced salt solution (BSS) supplemented with 2% heat-inactivated fetal calf serum, penicillin (100 U/ml), and streptomycin (100 μg/ml). Recipient rats were given $6 \times 10^7$ viable nucleated syngeneic bone marrow cells in 1.0 ml BSS intravenously into a tail vein. CsA, a kind gift from Sandoz Pharma Ltd. (Basel, Switzerland), was dissolved in olive oil at a concentration of 7.5 mg/ml. Rats received 7.5 mg CsA/kg-day, administered subcutaneously for 28 days.

### Thymectomies

While under ketamin and xylazine anesthesia, rats were intubated and maintained on a respirator. The thorax was opened by mediastinal incision and the thymus was entirely removed.

### Experimental Design

In the first experiment, the immunohistochemistry of the thymus was studied. Rats treated according to the previously mentioned protocol (SYN+) were compared to three different age-matched control (AMC) animals: rats irradiated and syngeneic bone marrow reconstituted and treated with olive oil only (SYN−), rats only treated with CsA (AMC+), and rats administered olive oil only (AMC−) (Table 2). Two rats from each group were sacrificed at 48 hr after irradiation and consecutive BMT and thereafter weekly starting from day 7 for up to 8 weeks.

TABLE 2

| | Irradiation | Syngeneic BMT | Cyclosporin A |
|---|---|---|---|
| SYN+ | + | + | + |
| SYN− | + | + | − |
| AMC+ | − | − | + |
| AMC− | − | − | − |

In the second experiment, the thymocyte population was studied by flow cytometry. Rats were treated similarly. The thymuses were collected at the same time points and prepared for flow cytometry.

The third experiment was designed to evaluate the disease-inducing effects of the separate treatments in combination with thymectomies. Thymectomies were performed on three different time points (days 7, 10, and 14) after irradiation and consecutive BMT (SYN− rats) or directly after cessation of a 4-week course of CsA administration (AMC+ rats).

### Antibodies

The mouse anti-rat monoclonal antibodies (mAb) used for immunohistochemistry are specified in Table 3. 1A29 was kindly provided by Prof. M. Miyasaka (Tokyo). The ED series was kindly provided by Dr. C.D. Dijkstra (Amsterdam). The set of HIS mAbs was kindly provided by Dr. J. Kampinga (Groningen, the Netherlands). W3/25 and the OX mAbs were obtained from the European Collection of Animal Cell Cultures (Salisbury, UK). OX62 was kindly provided by Dr. M. Brenan (Oxford), and R73 was kindly provided by Prof. Th. Hünig (Würzburg, Germany). RCK102 was obtained from Organon Teknika (the Netherlands).

### Immunohistochemistry

The thymus was snap frozen in isopentane and used for cryosections. Four- to six-μm-thick thymus cryosections were cut, air dried, and stored at −20°C. All thymus sections were stained using a two-step immunoperoxidase technique after acetone fixation. Sections were incubated for 60 min with mAb in the appropriate dilution. After washing in PBS, slides were incubated with a 1:200 dilution of rabbit anti-mouse Ig peroxidase conjugate (Dako, Denmark) in PBS with 0.5% bovine serum albumin (w/v) (BSA, Sigma) and 3% normal rat serum (v/v) for 30 min. After washing with PBS, the sections were stained for peroxidase activity with 3,3'-diaminobenzidine-tetrahydrochloride (Sigma, St. Louis) (0.5 mg/ml, in Tris-HCI buffer (pH 7.6), containing 0.02% (v/v) $H_2O_2$). Control sections were incubated in the same way omitting the first-step mAb. Subsequently, the sections were counterstained with hematoxylin, dehydrated, and covered with Entallan (Merck, Germany).

### Flow Cytometry

The thymus was teased apart and passed through a 100-μ-mesh nylon gauze and collected in BSS supplemented with 2% heat-inactivated new born calf

TABLE 3

| mAb | Antigen and Expression in the Thymus | Reference |
|---|---|---|
| 1A29 | ICAM-1, present on IDC and epithelium | Tamatani and Miyasaka, 1990 |
| ED1 | Macrosialin, present in Mϕ and IDC | Dijkstra et al., 1985 |
| ED2 | Cortical Mϕ | Dijkstra et al., 1985 |
| ED8 | Complement receptor type 3, present on IDC, Mϕ and granulocytes | Damoiseaux et al., 1989 |
| ED19 | CTES III[a] | |
| ED20 | CTES II | |
| HIS37 | CTES I | Kampinga et al., 1987 |
| HIS38 | CTES III | Kampinga et al., 1987 |
| HIS39 | CTES II | Kampinga et al., 1987 |
| HIS45 | Medullary thymocytes | Aspinall and Kampinga, 1989 |
| OX6 | MHC class II (RT1B), present on IDC, epithelium, and Mπ | McMaster and Williams, 1979 |
| OX8 | CD8, present on all cortical and a subset of the medullary thymocytes | Brideau et al., 1990 |
| OX18 | MHC class I (RT1A), present on all cells with high expression on epithelium, Mϕ, and IDC | Fukumoto et al., 1982 |
| OX19 | CD5, pan T-cell marker present on all thymocytes | Dallman et al., 1984 |
| OX39 | Interleukin-2 receptor, present on T blasts and IDC | Paterson et al., 1987 |
| OX62 | Integrin-like structure, present on IDC | Brenan and Puklavec, 1992 |
| R73 | T-cell receptor αβ | Hünig et al., 1989 |
| RCK102 | All types of keratin, not species-specific | |
| W3/35 | CD4, present on all cortical thymocytes, a subset of medullary thymocytes and Mϕ | Williams et al., 1977 |

[a] CTES: cluster of thymic epithelial staining
CTES I Pan epithelial cells
CTES II Subscapsular/perivascular and the majority of medullary epithelail cells
CTES III Cortical epithelial cells including thymic nurse cells (TNC)
CTES IV Hassall's corpuscles and all medullary epithelial cells
CTES IV Hassall's corpuscles and, sometimes, a surrounding halo of medullary epithelial cells serum. These cells were kept on ice until all samples were collected. The thymocytes were spun down (316 g, 10 min, 4°C), resuspended in PBS containing 0.5% BSA, and counted in a Bürker Hemocytometer while viability was assessed by trypan blue exclusion. For tricolor flow cytometry, $5 \times 10^5$ cells per sample were centrifuged in a 96-well microtiter plate (236 g, 3 min, 4°C) and resuspended in 20 µl PBS containing 0.5% BSA, 10 mM $NaN_3$, and the following conjugated mAbs: ER2 (Joling et al., 1985) conjugated to FITC (a kind gift from Dr. B. de Geus, Leiden, the Netherlands) recognizing the rat CD4 antigen, OX8 conjugated to PE (Caltag, Hycult, Uden, the Netherlands) recognizing the rat CD8 antigen, and R73 conjugated to biotin (a kind gift from Prof. Th. Hünig, Würzburg) recognizing the rat T-cell receptor αβ chain (TCRαβ) and that is detected in a second-step labeling by the streptavidin conjugated fluorochrome Cy-Chrome (Pharmingen, USA). By using these three labeled mAbs together with the appropriate controls and by using optimal settings and compensation, the expression of CD4, CD8, and TCRαβ was determined on a FACSort (Becton Dickinson).

### ACKNOWLEDGMENTS

The authors wish to thank Prof. Th. Hünig (Würzburg, Germany), Prof. M. Miyasaka (Tokyo), Dr. C.D. Dijkstra (Amsterdam), Dr. J. Kampinga (Groningen, the Netherlands), Dr. M. Brenan (Oxford), and Dr. B. de Geus (Leiden, the Netherlands) for the generous gift of monoclonal antibodies. Additionally, we wish to thank Prof. Dr. A. Kruisbeek and Dr. L. Jones (Netherlands Cancer Institute, Amsterdam) for their help in developing the rat tricolor flow cytometry, and Prof. Dr. J. Raus and J. Lambrechts (Dr. L. Willems Institute, Diepenbeek, Belgium) for providing their facilities to analyze our flow cytometry samples. Furthermore, we would like to express our gratitude toward Mrs. M.-J. van de Gaar and M. Vaessen for their excellent technical assistance. This project was financed by the Dutch Organization for Scientific Research (Grant: 900-506-160).

*(Received February 23, 1994)*

*(Accepted June 1, 1994)*

### REFERENCES

Aspinall R., and Kampinga J. (1989). A novel lymphocyte surface antigen recognized by the monoclonal antibody HIS45. Thymus **13**: 245–252.

Beschorner W.E., and Armas O.A. (1991a). Loss of medullary dendritic cells in the thymus after cyclosporine and irradiation. Cell. Immunol. **132**: 505–514.

Beschorner W.E., Di Gennaro K.A., Hess A.D., and Santos G.W. (1987). Cyclosporine and the thymus: Influence of irradiation and age on thymic immunopathology and recovery. Cell Immunol. **110**: 350–364.

Beschorner W.E., Hess A.D., Shinn C.A., and Santos G.W. (1988a). Transfer of Cyclosporine-associated syngeneic graft-versus-host disease by thymocytes. Transplantation **45**: 209–215.

Beschorner W.E., Olson J.L., Hess A.D., Di Gennaro K.A., and Santos G.W. (1988b). Cyclosporine-induced cell-mediated injury of the thymic medullary epithelium. Transplantation **45**: 797–803.

Beschorner W.E., Ren H., Phillips J., Pulido H.B.J., Hruban R.H., and Hess A. D. (1991b). Prevention of syngeneic graft-versus-host disease by recovery of thymic microenvironment after cyclosporine. Transplantation **52**: 668–674.

Bos G.M.J., Majoor G.D., and van Breda Vriesman P.J.C. (1988). Cyclosporin-A induces a selective, reversible suppression of T-helper lymphocyte regeneration after syngeneic bone marrow transplantation: Association with syngeneic graft-versus-host disease. Clin. Exp. Immunol. **74**: 443–448.

Brenan M., and Puklavec M. (1992). The MRC OX-62 antigen: A useful marker in the purification of rat veiled cells with the biochemical properties of an antigen. J. Exp. Med. **175**: 1457–1465.

Brideau R.J., Carter P.B., McMaster W.R., Mason D.W., and Williams A.F. (1980). Two subsets of rat T lymphocytes defined with monoclonal antibodies. Eur. J. Immunol. **10**: 609–615.

Bruijntjes J.P., Kuper F., Robinson J.E., and Schuurman H.-J. (1993). Epithelium free area in the thymic cortex of rats. Dev. Immunol. **3**: 113–122.

Bryson J.S., Caywood B.E., and Kaplan A.M. (1991). Relationship of cyclosporin A-mediated inhibition of clonal deletion and development of syngeneic graft-versus-host disease. J. Immunol. **147**: 391–397.

Cheney R.T., and Sprent J. (1985). Capacity of Cyclosporin to induce auto-graft-versus-host disease and impair intrathymic T cell differentiation. Transplant. Proc. **17**: 528–530.

Dallman M.J., Thomas M.L. and Green J.R. (1984). MRC OX-19: A monoclonal antibody that labels rat T lymphocytes and augments in vitro proliferative responses. Eur. J. Immunol. **14**: 260–267.

Damoiseaux J.G.M.C., Beijleveld L.J.J., and van Breda Vriesman P.J.C. (1993). Quantification and phenotypic characterization of the rat thymic dendritic cell population upon in vivo cyclosporine administration. Transplant. Proc. **25**: 2814–2815.

Damoiseaux J.G.M.C., Döpp E.A., Neefjes J.J., Beelen R.H.J., and Dijkstra C.D. (1989). Heterogeneity of macrophages in the rat evidenced by variability in determinants: Two new anti-rat macrophage antibodies against a heterodimer of 160 and 95 kd (CD11/CD18). J. Leukocyte Biol. **46**: 556–564.

De Waal E.J., Rademakers L.H.P.M., Schuurman H.J., and Van Loveren H. (1992). Interdigitating cells in the rat thymus during cyclosporin A treatment: Ultrastructural observations. Thymus **20**: 163–170.

Dijkstra C.D., Döpp E.A., Joling P., and Kraal G. (1985). The heterogeneity of mononuclear phagocytes in lymphoid organs: Distinct macrophage subpopulations in the rat recognized by monoclonal antibodies ED1, ED2 and ED3. Immunology **54**: 589–599.

Fukumoto, T., McMaster W.R., and Williams A.F. (1982). Mouse monoclonal antibodies against rat major histocompatibility antigens. Two Ia antigens and expression of Ia and class I antigens in rat thymus. Eur. J. Immunol. **12**: 237–243.

Gao E.K., Lo D., Cheney R., Kanagawa O., and Sprent J. (1988).

Abnormal differentiation of thymocytes in mice treated with cyclosporin A. Nature **336**: 176–179.

Glazier A., Tutschka P.J., Farmer E.R., and Santos G.W. (1983). Graft-versus-host disease in cyclosporin A-treated rats after syngeneic and autologous bone marrow reconstitution. J. Exp. Med. **158**, 1–8.

Hünig T., Wallny H.-J., Hartley J.K., Lawetzky A., and Tiefenthaler G. (1989). A monoclonal antibody to a constant determinant of the rat T cell antigen receptor that induces T cell activation. J. Exp. Med. **169**: 73–86.

Jenkins M.K., Schwartz R.H., and Pardoll D.M. (1988). Effects of Cyclosporin A on T cell development and clonal deletion. Science **241**: 1655–1658.

Joling P., Tielen F.J., Vaessen L.M.B., Huijbregts J.M.A., and Rozing J. (1985). New markers on T cell subpopulations defined by monoclonal antibodies. Transplant Proc. **17**: 1857–1860.

Jones R.J., Hess A.D., Mann R.B., Piantadosi S., Vogelsang G.B., Farmer E.R., Geller R.B., and Santos G.W. (1989). Induction of graft-versus-host-disease after autologous bone marrow transplantation. Lancet **1**: 754–757.

Kampinga J., Kroese F.G.M., Duijvestijn A.M., Murawska M.B., Pol G.H., and Nieuwenhuis P. (1987). The rat thymus microenvironment: Subsets of thymic epithelial cells defined by monoclonal antibodies. Transpl. Proc. **19**: 3171–3174.

Kanariou M., Huby R., Ladyman H., Colic M., Sivolapenko G., and Lampert I. (1989). Immunosuppression with cyclosporin A alters the thymic microenvironment. Clin. Exp. Immunol. **78**: 263–270.

McCarthy S.A., Cacchione R.N., Mainwaring M.S., and Cairns J.C. (1992). The effects of immunosuppressive drugs on the regulation of activation-induced apoptotic cell death in thymocytes. Transplantation **54**: 543–547.

McMaster W.R., and Williams A.F. (1979). Identification of Ia glycoproteins in rat thymus and purification from rat spleen. Eur. J. Immunol. **9**: 426–433.

Mulder A.H., and Visser J.W.M. (1988). The entry of the prothymocyte into the thymus after lethal irradiation and bone marrow transplantation. I. Seeding of bone marrow cells into the thymus. Thymus **11**: 15–27.

Mulder A.H., Visser J.W.M., Zoetelief J., and van Bekkum D.W. (1988). The entry of the prothymocyte into the thymus after lethal irradiation and bone marrow transplantation. II. Time of entry. Thymus **11**: 29–41.

Murawska M.B., Duijvestijn A.M., Klatter F.A., Ammerlaan W., Meedendorp B., and Nieuwenhuis P. (1991). Differential kinetics of various subsets of thymic bone marrow–derived stromal cells in rat chimeras. Scand. J. Immunol. **33**: 473–484.

Paterson D.J., Jefferies W.A., Green J.R., Brandon M.R., Corthesy P., Puklavec M., and Williams A.F. (1987). Antigens of activated rat T lymphocytes including a molecule of 50,000 Mr detected only on CD4 positive T blasts. Mol. Immunol. **24**: 1281–1290.

Ritter M.A., and Boyd R.L. (1993). Development in the thymus: It takes two to tango. Immunol. Today **14**: 462–469.

Ritter M.A, and Ladyman H.M. (1991). The effects of cyclosporin on the thymic microenvironment and T cell development. In Thymus Update, Kendall M.D., and Ritter, M.A., Eds. (Chur: Harwood Academic Publishers) pp. 157–177.

Schreiber S.L., and Crabtree G.R. (1992). The mechanism of action of cyclosporin A and FK506. Immunol. Today **13**: 136–141.

Schuurman H.-J., van Loveren H., Rozing J., van Dijk A., Loeber J.G., and Vos J.G. (1990). Cyclosporin and the rat thymus. An immunohistochemical study. Thymus **16**: 235–254.

Shores E.W., van Ewijk W., and Singer A. (1991). Disorganization and restoration of thymic medullary epithelial cells in T-cell receptor-negative SCID mice: Evidence that receptor-bearing lymphocytes influence maturation of the thymic microenvironment. Eur. J. Immunol. **21**: 1657–1661.

Sorokin R., Kimura H., Schroder K., Wilson D.H., and Wilson D.B. (1986). Cyclosporin-induced autoimmunity: Conditions for expressing disease, requirement for intact thymus, and potency estimates of autoimmune lymphocytes in drug-treated rats. J. Exp. Med. **164**: 1615–1625.

Tamatani T., and Miyasaka M. (1990). Identification of monoclonal antibodies reactive with the rat homolog of ICAM-1, and evidence for a differential involvement of ICAM-1 in the adherence of resting versus activated lymphocytes to high endothelial cells. Int. Immunol. **2**: 165–171.

Tanaka M., Shihonara K., Fukumoto T., Tanaka H., and Kaneko T. (1988). Effect of Cyclosporin A on rat thymus: Time course analysis by immunoperoxidase technique and flow cytometry. Clin. Exp. Immunol. **72**: 216–221.

Thomson A.W. (1992). The spectrum of action of new immunosuppressive drugs. Clin. Exp. Immunol. **89**: 170–173.

Williams A.F., Galfre G., and Milstein C. (1977). Analysis of cell surfaces by xenogeneic myeloma-hybrid antibodies: Differentiation antigens of rat lymphocytes. Cell **12**: 663–000.

Wodzig K.W.H., Majoor G.D., and van Breda Vriesman P.J.C. (1993). Kinetics of inducer/effector cell generation in the thymus in cyclosporine-induced autoimmunity. Transplant. Proc. **25**: 2819–2821.

Yasutomi D., Odaka C., Saito S., Niizeki H., Kizaki H., and Tadakuma T. (1992). Inhibition of programmed cell death by cyclosporin A: Preferential blocking of cell death induced by signals via TCR/CD3 complex and its mode of action. Immunology **77**: 68–74.

